# Leptospiral Immunoglobulin-Like Domain Proteins: Roles in Virulence and Immunity

**DOI:** 10.3389/fimmu.2020.579907

**Published:** 2021-01-08

**Authors:** David A. Haake, James Matsunaga

**Affiliations:** ^1^ Division of Infectious Diseases, VA Greater Los Angeles Healthcare System, Los Angeles, CA, United States; ^2^ Departments of Medicine, and Microbiology, Immunology, and Molecular Genetics, David Geffen School of Medicine, University of California, Los Angeles, CA, United States; ^3^ Research Service, VA Greater Los Angeles Healthcare System, Los Angeles, CA, United States; ^4^ Department of Medicine, David Geffen School of Medicine, University of California, Los Angeles, CA, United States

**Keywords:** leptospira, leptospirosis, virulence, vaccine, adhesin, complement resistance, immunoglobulin-like domain, hemostasis

## Abstract

The virulence mechanisms required for infection and evasion of immunity by pathogenic *Leptospira* species remain poorly understood. A number of *L. interrogans* surface proteins have been discovered, lying at the interface between the pathogen and host. Among these proteins, the functional properties of the Lig (leptospiral immunoglobulin-like domain) proteins have been examined most thoroughly. LigA, LigB, and LigC contain a series of, 13, 12, and 12 closely related domains, respectively, each containing a bacterial immunoglobulin (Big) -like fold. The multidomain region forms a mostly elongated structure that exposes a large surface area. Leptospires wield the Lig proteins to promote interactions with a range of specific host proteins, including those that aid evasion of innate immune mechanisms. These diverse binding events mediate adhesion of *L. interrogans* to the extracellular matrix, inhibit hemostasis, and inactivate key complement proteins. These interactions may help *L. interrogans* overcome the physical, hematological, and immunological barriers that would otherwise prevent the spirochete from establishing a systemic infection. Despite significant differences in the affinities of the LigA and LigB proteins for host targets, their functions overlap during lethal infection of hamsters; virulence is lost only when both *ligA* and *ligB* transcription is knocked down simultaneously. Lig proteins have been shown to be promising vaccine antigens through evaluation of a variety of different adjuvant strategies. This review serves to summarize current knowledge of Lig protein roles in virulence and immunity and to identify directions needed to better understand the precise functions of the Lig proteins during infection.

## Introduction

Leptospiral immunoglobulin-like (Lig) domain proteins play key roles in the pathogenesis mechanisms of the spirochetes responsible for leptospirosis, an infectious disease that is re-emerging worldwide. The widespread dissemination of leptospires is related to their ability to colonize the renal tubules of a wide variety of mammalian hosts. Some mammals are reservoir hosts that do not exhibit signs of disease but remain colonized for extended periods of time, during which they shed the spirochetes in their urine and contaminate the surrounding environment. Leptospires may remain viable for extended periods of time in moist soil or freshwater until infecting a new host. Infection is initiated through penetration of skin abrasions and mucous membranes and widespread hematogenous dissemination. The ensuing host inflammatory response results in either clearance of infection, selective colonization of the renal tubules, or multiorgan system failure and death.

To date, 64 leptospiral species have been described, which are distributed into 4 clades: P1, P2, S1, and S2 (1). The clade designation is preferred to the older pathogen, intermediate, and saprophytic designations because the virulence status of many of these species is unknown. Clades P1 and P2 include species known to cause human infections (1). *L. interrogans* is the most prevalent cause of severe human infection and the subject of most studies on the molecular basis of leptospiral pathogenesis. We are only now beginning to unravel the mechanisms by which *L. interrogans* infects susceptible hosts and causes disease. Key to *L. interrogans* survival in the host during the early phases of infection is evasion of the innate immune response. Elimination of *L. interrogans* requires recognition of microbe-associated molecular patterns (MAMPs) by pattern recognition receptors (PRRs) including members of the Toll-like receptor (TLR) and NOD-like receptor (NLR) families (2). Leptospires must evade the bactericidal effects of complement (3), avoid becoming entrapped in fibrin mesh, and escape capture by neutrophil extracellular traps (NETs), phagocytic activity, and antimicrobial components (4). By the time the humoral immune response produces antibodies targeting *L. interrogans*, the spirochetes have disseminated to various organs including the kidneys, liver, and other organs and triggered the release of pro-inflammatory cytokines (5).

Among the potential leptospiral surface proteins that have been identified as adhesins based on *in vitro* adherence studies, the Lig proteins stand out in terms of the range of evidence supporting their role in virulence. Not only have the Lig proteins been found to adhere strongly to a surprising variety of host ligands, indicating a unique level of multifunctionality, gain of function adherence studies have been reported using the normally non-adherent saprophyte, *L. biflexa* (6–8). Some of the host ligands targeted by the Lig proteins indicate a role for the Lig proteins in evasion of innate immunity. The Lig proteins are strongly upregulated in response to host temperature and osmolarity conditions (9–11), indicating a role in infection of the mammalian host. Virulence is lost when transcription of both *ligA* and *ligB* is knocked down, indicating that expression of at least one *lig* gene is required for infection. The Lig proteins are some of the earliest proteins identified by the humoral immune response to leptospirosis and are among the strongest immunogens. Purified recombinant Lig proteins function as potent immunoprotective antigens in animal models of leptospirosis. This review will describe what is known about the role of the Lig family of proteins in the molecular pathogenesis of *L. interrogans*. Here we present an integrated model of the role of Lig proteins in leptospiral virulence and immunity. This is the first focused review on the Lig proteins that we are aware of.

## Discovery and Description

The *ligA* gene was first described by Palaniappan *et al*. in an equine strain of *L. interrogans* serovar Pomona, subtype kennewicki (12). The gene was found by screening a bacteriophage expression library with serum from a mare after a leptospiral infection that had induced abortion. Sequencing of one positive clone revealed a large open reading frame encoding a protein with a calculated molecular mass of ~130 kDa. They noted a series of tandem 90 amino acid imperfect repeat domains, each predicted to fold into an immunoglobulin-like domain. This observation suggested a virulence function for LigA because the well-characterized bacterial adhesins *Yersinia* invasin and *E. coli* intimin also harbored multiple Ig-like domains. The remaining members of the *lig* gene family were described in a study published a year later (13) using pooled sera from leptospirosis patients to identify the *ligA*, *ligB*, and *ligC* genes in *L. interrogans* and *L. kirschneri* expression libraries.

Lipoprotein signal peptide sequences present at the amino termini of the Lig proteins suggested that these were exported proteins. Lipidation occurred during incubation of *E. coli* expressing LigA in tritiated palmitate (14). Export to the leptospiral surface has been demonstrated by whole cell and thin section immunoelectron microscopy (13). Surface exposure has been confirmed by susceptibility of LigA of intact *L. interrogans* to proteinase K digestion (14) and by immunofluorescence microscopy using LigB antiserum (10). Taken together, this evidence indicates that the Lig proteins are exported to the outer membrane in a lipidated form by the localization of lipoprotein (Lol) pathway and then translocated to the leptospiral surface (15).

Selective extracellular release of LigA but not LigB, into the culture supernatant was observed with *L. kirschneri* and *L. interrogans* (10, 11). Released LigA was in a truncated form, suggesting involvement of proteolysis in its release. Soluble LigA levels were minimal with *L. kirschneri* at exponential growth phase in EMJH (standard growth medium) and accumulated in stationary phase as cellular LigA levels decreased (10). With *L. interrogans*, levels of both cellular and soluble LigA increased considerably when sodium chloride was added to achieve the osmolarity of mammalian tissues. However, when the osmolarity was increased with sucrose or glucose, soluble LigA was not detected despite the increased levels of cellular LigA (11). This raises the possibility that glucose, a component of blood, inhibits proteolysis of LigA in the bloodstream. Soluble LigA may have immunomodulatory properties that aid survival of *Leptospira* during infection, as demonstrated with proteins released from the surface of other bacteria (16).

**Figure 1:**
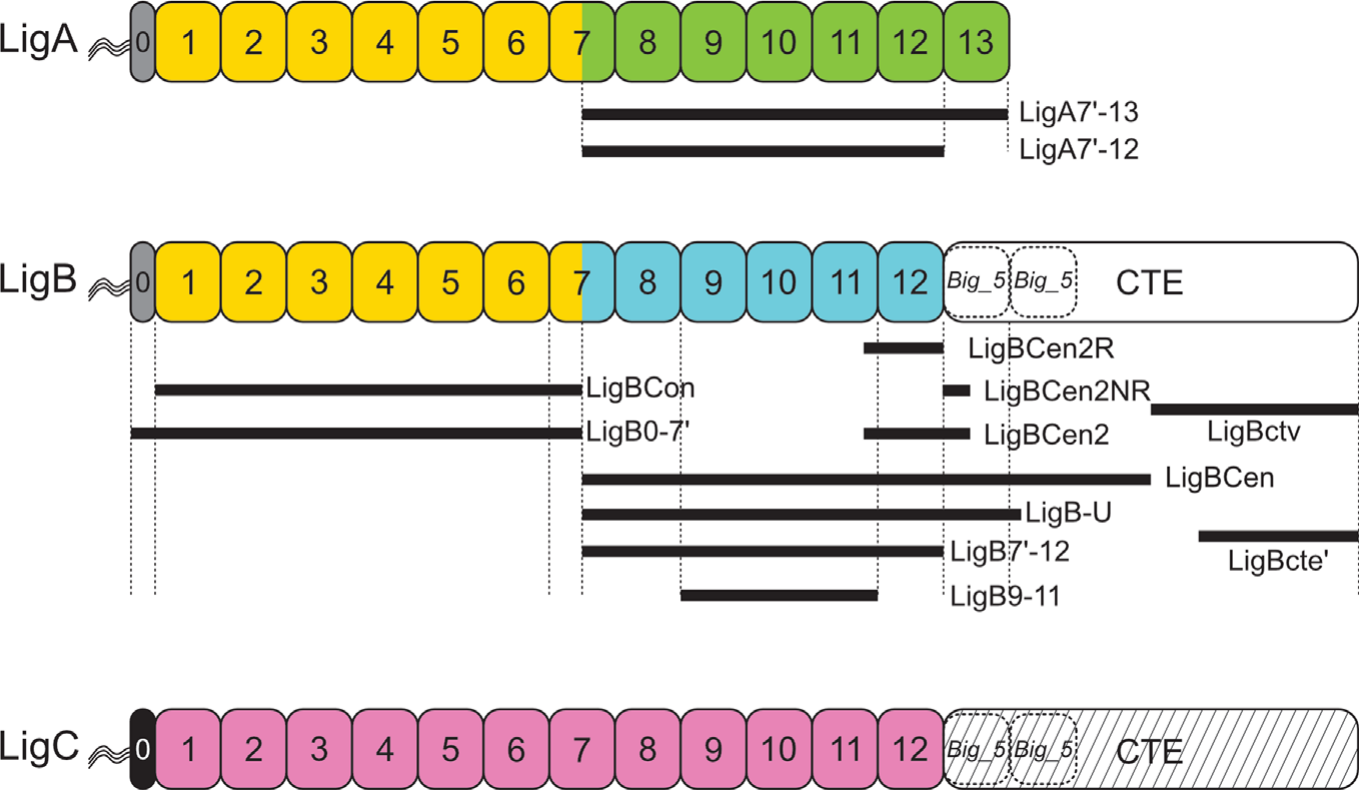
Leptospiral Immunoglobulin-Like (Lig) Protein Immunoglobulin-like Domain Structure. Domain structures of the three Lig proteins are shown. LigA, LigB, and LigC contain a series of 13, 12, and 12 Big_2 domains, respectively. The first six Big_2 domains and part of the seventh domain of LigA and LigB have a high percentage of sequence identity as indicated by yellow domains. The sequences of the remaining Big_2 domains differ between LigA (green) and LigB (blue). The Big_2 domains of LigC are shown in pink. The Big_2 domains of LigB and LigC are followed by a C-terminal extension (CTE) predicted to harbor Big_5 domains (dashed rectangle). The “0” segment between the amino terminus of the mature proteins and Big_2 domain 1 is indicated with gray or black shading. All three Lig proteins are thought to have triacylated amino-terminal cysteines. Lig fragments are shown with black lines, and their amino acid coordinates are: LigA7’-13 (631–1224), LigBCon (47-630), LigBCen (631-1417), LigBCen2R (1014-1124), LigBCen2NR (1120-1165), LigBCen2 (1014-1165), LigBCen2NR (1014-1124), LigBCen2NR (1120-1165), LigB7’-12 (631-1124), LigB-U (631-1257), LigBctv (1418-1889), LigBcte’ (1630-1890). Lig fragment nomenclature may differ from that used in the original studies.

**Figure 2:**
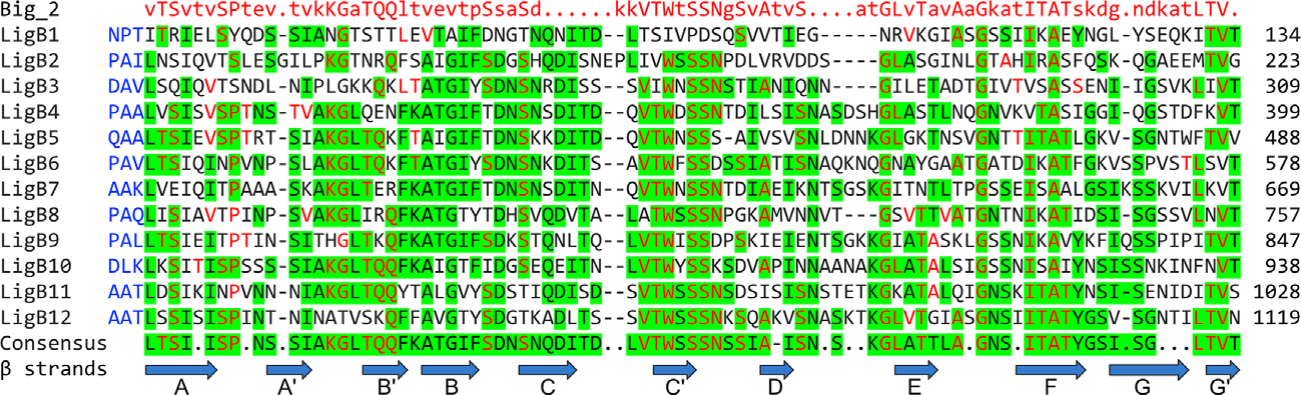
Leptospiral Immunoglobulin-Like B (LigB) Domain Alignment. Alignment of the 12 Big_2 domains of *L. interrogans* serovar Copenhageni strain Fiocruz L1-130 is shown, green highlights indicate regions of consensus among the domains. Red font indicates agreement with the Big_2 domain consensus sequence shown in the top row. Blue font indicates sequence of 3 amino acid linkers between LigB domains. Amino acid position of the terminal amino acid in each domain is provided on the right. Blue arrows indicate regions of beta sheet in the LigB12 solution structure (19).

LigA, LigB, and LigC contain a series of 13, 12, and 12 immunoglobulin-like (Ig-like) fold domains, respectively, belonging to the Big_2 domain protein family PF02368 (**Figure 1**). Big_2 protein domains are widely distributed in nature including many bacterial species, including host cell binding proteins such as the *E. coli* intimin host cell-adhesion molecule (17) and phage tail proteins (18). The first six and part of the seventh Ig-like domains of LigA and LigB are nearly identical between these two proteins, while the remaining domains are unique to each protein. While all the Lig protein Ig-like domains belong to the Big_2 protein family and their sequences are related, they are not “repeats” and share only a moderate degree of amino acid sequence identity (**Figure 2**). Adjacent Ig-like domains are connected by three amino acid “linkers” that separate domains and may enable them to flex relative to one another (**Figure 2**). As shown in **Figure 1**, the 12 Ig-like domains of LigB are followed by two putative Big_5 domains that are part of a large carboxyterminal extension (CTE) of 772 amino acids that is not present in LigA. These two Big_5 domains are identified by a sequence search of the Pfam database version 33.1 (https://pfam.xfam.org/) (20) using the LigB CTE as the query sequence. LigC is organized similarly to LigB in that both proteins contain 12 Ig-like domains followed by two Big_5 domains as part of a large CTE. The amino acid sequence identity of the LigB and LigC CTEs is 51% (13).

The large CTEs of LigB and LigC are likely to be important for the structure and function of these proteins. A complete understanding of LigB structure and how the mature protein is integrated into the leptospiral outer membrane is lacking without a better understanding of the structure and function of the CTE.

## Structure

The Lig protein domains feature the immunoglobulin-like fold, which was initially described for an Fab’ fragment of human immunoglobulin in 1973 (21). The subsequent discovery of the immunoglobulin fold in numerous proteins functionally unrelated to the immunoglobulins led to the description of the immunoglobulin superfamily, which is defined by its characteristic fold rather than its primary sequence (22). The classic Ig-fold comprises 70 to 100 amino acid residues divided into two stacked β sheets with a total of at least seven and as many as ten anti-parallel β strands (23). The Ig-fold is divided into subtypes based on their topology and sequence (24). A comparison of the first 52 known structures of the fold revealed that in most cases strands A, B, and E were located in the first β sheet and strands C, F, and G in the second sheet (24). Depending on the subtype, the first β sheet may also have strand D, and the second sheet may have one, two, or all of strands A’, C’, and C’’ (23). Ig-like domains within each subtype share a hydrophobic core comprising several hydrophobic residues that have their side chains directed toward the interior of the domain, primarily from strands B, C, E, and F. These proteins are found in all domains of life and both inside and outside the cell (24).

**Figure 3:**
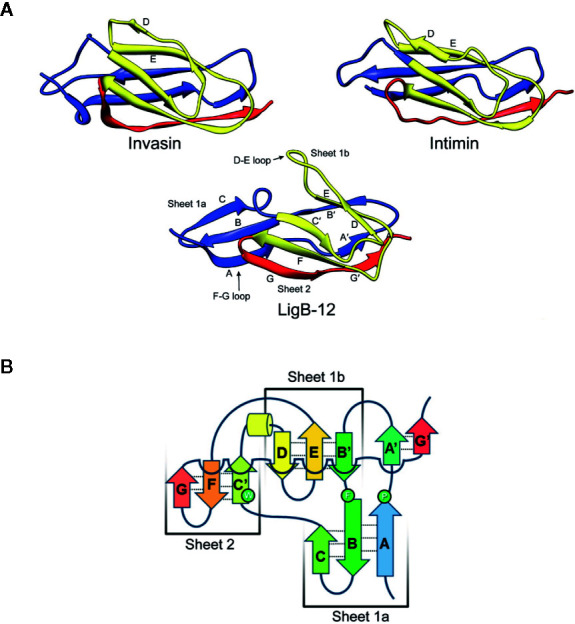
Comparison of Leptospiral Immunoglobulin-Like B (LigB) Domain 12 and Invasin Domain 2 Structures. **(A)** The most stable NMR solution structures of LigB12 (PDB entry: 2MOG) from *L. interrogans* serovar Pomona type kennewicki strain JEN4 (26), and the D2 domain of invasion (PDB entry: 1CWV) from *Yersinia pseudotuberculosis* (27) are presented showing the immunoglobulin-like fold. LigB12 is composed of a β sandwich with ten β strands distributed over the two layers, with the more extensive divided into two smaller sheets. Invasin is composed of a β sandwich with ten β strands in two sheets. Note the strands extending from the amino- and carboxy-termini of the domain, which serve as linkages between domains. The images were obtained from the RCSB Protein Data Bank (28). **(B)** The secondary structure of LigB12 is represented as a topology diagram (26). Images from Ptak *et al*., 2014 (https://pubs.acs.org/doi/10.1021/bi500669u) (26), with permission from the American Chemical Society.

The solution structures of domains LigA4 and LigB12 were solved by NMR (19, 25, 26). Both domains adopt a similar Ig-like fold; however, their structures differ from the classic two-layer sandwich described for other Ig-like domains, such as the D2 domain of invasin from *Yersinia pseudotuberculosis* (**Figure 3A**) (27). **Figure 3A** shows the structure of LigB12. The Ig-like fold of both LigA4 and LigB 12 consists of 10 β-strands divided into one large β sheet, sheet 2 (β strands C’, F, and G), and two smaller sheets, 1a (strands A, B, and C) and 1b (strands B’, D, and E), with β strand B/B’ split between sheets 1a and 1b (**Figure 3B**). β sheets 1b and 2 form the β sandwich, while sheet 1a extends beyond the rest of the domain to leave both of its surfaces solvent-exposed (**Figure 3A**). The residues directed toward the hydrophobic core are highly conserved among the Lig Ig-like domains (26). The Ig-like domains of LigA and LigB lack the cysteines that connect the β sheets of some Ig domains with a disulfide bridge to make the core more compact (26).

To obtain the low-resolution structure of the 12 LigB domains in series, a series of LigB polypeptides comprising a sliding window of five consecutive Ig-like domains covering all 12 LigB Ig-like domains were subjected to small angle X-ray scattering (SAXS) (29). The tandem Ig-like domains of LigB adopts an elongated conformation without being fully extended. Kinks occur at domains 2 and 7, and the structure beyond domain 7 forms a gentle spiral. Interdomain interactions between neighboring domains may occur *via* a salt bridge between lysine and aspartate residues in adjacent domains (26). The lysine and aspartate residues are found at the same position in most of the Ig-like domains of LigA and LigB. On the other hand, only three of the twelve domains of LigC have lysine at the equivalent position, although 11 of the 12 have the aspartate residue. Salt bridges are unlikely to contribute significantly to the kinks in the extended structure of the Lig proteins since the location of paired lysine and arginine residues in adjacent domains do not correlate with the location of the kinks. However, the salt bridges likely hinder rotation of neighboring domains. The low sequence identity between adjacent Lig Ig-like domains may be necessary to prevent interdomain misfolding following its biosynthesis (30).

LigA and LigB are calcium-binding proteins (31). An early study showed that LigBCen2, which comprised LigB12 plus extra amino acids at its amino- and carboxy termini, bound to calcium (32). It was shown later that individual Ig-like domains LigA9 and LigA10 bound to calcium, confirming that the residues involved in calcium binding were contained entirely within the Ig-like domains (31). LigA9, LigA10, and LigBCen2 formed homodimers in the absence of calcium (31). The Lig proteins were the first examples of the new class of Ig-like domains that bind calcium (33). LigA4, LigBCen2, and LigBCen2R (comprising LigB12 and an N-terminal extension, **Figure 1**) also bound with high affinity to Ca^2+^, and a chemical shift was observed in NMR experiments upon the addition of Ca^2+^ (19, 25, 32). However, this observation was not confirmed in a separate NMR study that showed no chemical shift of LigB12 when CaCl_2_ or EGTA were added (26).

## Evolution and Relatedness

**Figure 4:**
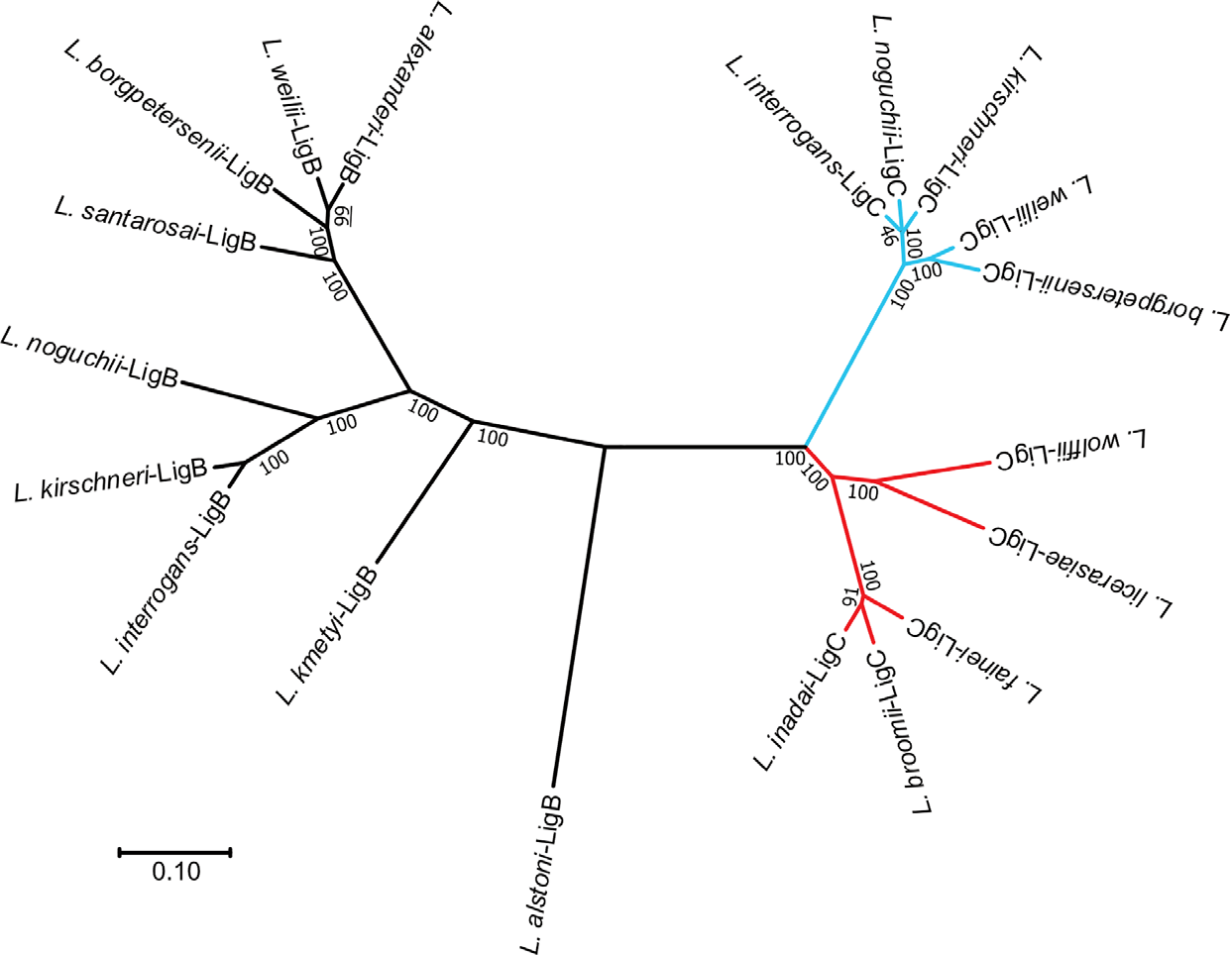
Phylogenetic Tree of Leptospiral Immunoglobulin-Like B (LigB) and LigC. An unrooted phylogenetic tree of the LigB and LigC proteins is shown. Sequences were aligned with MUSCLE (35), and the tree was generated with the Neighbor Joining distance algorithm. Branch lengths represent the distance between nodes. Scale bar shows the distance for a 10% difference between sequences. Bootstrap values from 1,000 replicates are shown. LigB branches are shown in black. Branches for clade P1 LigC proteins are indicated in light blue. Clade P2 LigC branches are colored red. Clade P1 sequences were obtained from *L. .interrogans* sv Lai str 56601 (AAN50976, AAN50273), *L. kirschneri* sv Grippotyphosa str. RM52 (EJO71150), *L. kirschneri* sv Cynopteri str 3522CT (EPG39682), *L. noguchii* sv Panama str CZ214T (EQA72229, EQA70476), *L. alstoni* sv Pingchang str 80-412 (EQA78456, EQA70476), *L. weilii* str Ecochallenge (EMY15141, EMY13008), *L. alexanderi* sv Manhao 3, str L 60T (EQA60934), *L. borgpetersenii* sv Javanica str UI 09931 (EPG5 EQA784568006, EPG56034), *L. santarosai* sv Shermani str 1342KT (EPG84276), and *L. kmetyi* sv Malaysia str Bejo-Iso9 (EQA54247). Clade P2 sequences were obtained from *L. fainei* sv Hurstbridge str BUT6T (EPG73553), *L broomii* sv Hurstbridge str 5399T (EQA44381), *L. wolffii* sv Khorat str Khorat-H2T (EPG64420), *L. licerasiae* sv Varillal str VAR 010 (EIE02109), and *L. .inadai* sv Lyme str 10T (EQA38254).

Whole-genome sequencing reveals that >90% leptospiral species in clades P1 and P2 have at least one member of the *lig* gene family (1). In Clade P1 species, the *ligB* gene is most consistently present, while the *ligA* and *ligC* genes are only present in selected species. The version of Lig protein present in Clade P2 species has been classified as LigC (34), though the phylogenetic tree of the Lig proteins in **Figure 4** shows that it is distinct from the LigC proteins found in *L. interrogans* and other Clade P1 species. Species in Clades S1 and S2 appear to lack *lig* genes. This suggests that an ancestral *lig* gene, similar in structure to *ligB* and *ligC*, was acquired after divergence of P and S clades and prior to divergence of Clades P1 and P2. Presumably, after divergence of Clade P1, duplication of this ancestral *lig* gene led to the formation of *ligB* and *ligC*. Subsequently, *ligA* was derived from *ligB* by gene duplication with loss of the carboxy-terminal region, as suggested by McBride et al. (36). The sequences of all three *lig* genes exhibit evidence of horizontal gene flow and recombinational events between *lig* genes of related *Leptospira* strains (36). Additional studies are needed to obtain a more accurate understanding of *lig* gene distribution and relatedness among leptospiral species.

In *L. interrogans* serovar Copenhageni strain Fiocruz L1-130 and *L. kirschneri* serovar Grippotyphosa strain RM52, the regions immediately upstream of the *ligA* and *ligB* coding regions are identical. In the Fiocruz L1-130 strain, the region of identity begins -266 nucleotides upstream of the *ligA* and *ligB* start codons and extends to position +1890 encoding the part of the seventh Ig-like domain with only minor coding differences between *ligA* and *ligB* (13). Inclusion of *E. coli*-like -35 and -10 RNA polymerase binding elements upstream of the transcriptional start site at position -175 as mapped by 5’ RACE (9) and confirmed by differential RNAseq designed to identify primary transcripts (37) suggests that *ligA* and *ligB* are transcriptionally co-regulated. Maintenance of this high degree of gene identity between *ligA* and *ligB* implies a gene conversion process involving replacement of this segment of one gene by the other. Gene conversion is likely facilitated by the arrangement of *ligA* and *ligB* as tandem genes on some leptospiral chromosomes.

## Virulence and Gene Regulation

There were clues that the Lig proteins were involved in pathogenesis even before the tools for the genetic manipulation of *L. interrogans* became available. The initial study on *ligA* described it as a gene that was induced during infection (12). LigA could not be detected in lysates of *L. interrogans* growing *in vitro*, yet antibody against LigA was detected in sera from infected mares, suggesting that expression of *ligA* was infection-associated. This was confirmed by reactivity of LigA antiserum with kidney sections from infected hamsters (12). Loss of virulence observed with multiple culture passage of *L. interrogans* is correlated by a reduction of LigA and LigB expression (13, 38). Subsequently it was found that LigA and LigB expression increased *in vitro* under growth conditions encountered in the host, when various salts were added to the culture medium to attain physiological osmolarity (10, 11) or when the culture temperature was increased to 37°C (9, 39).

**Figure 5:**
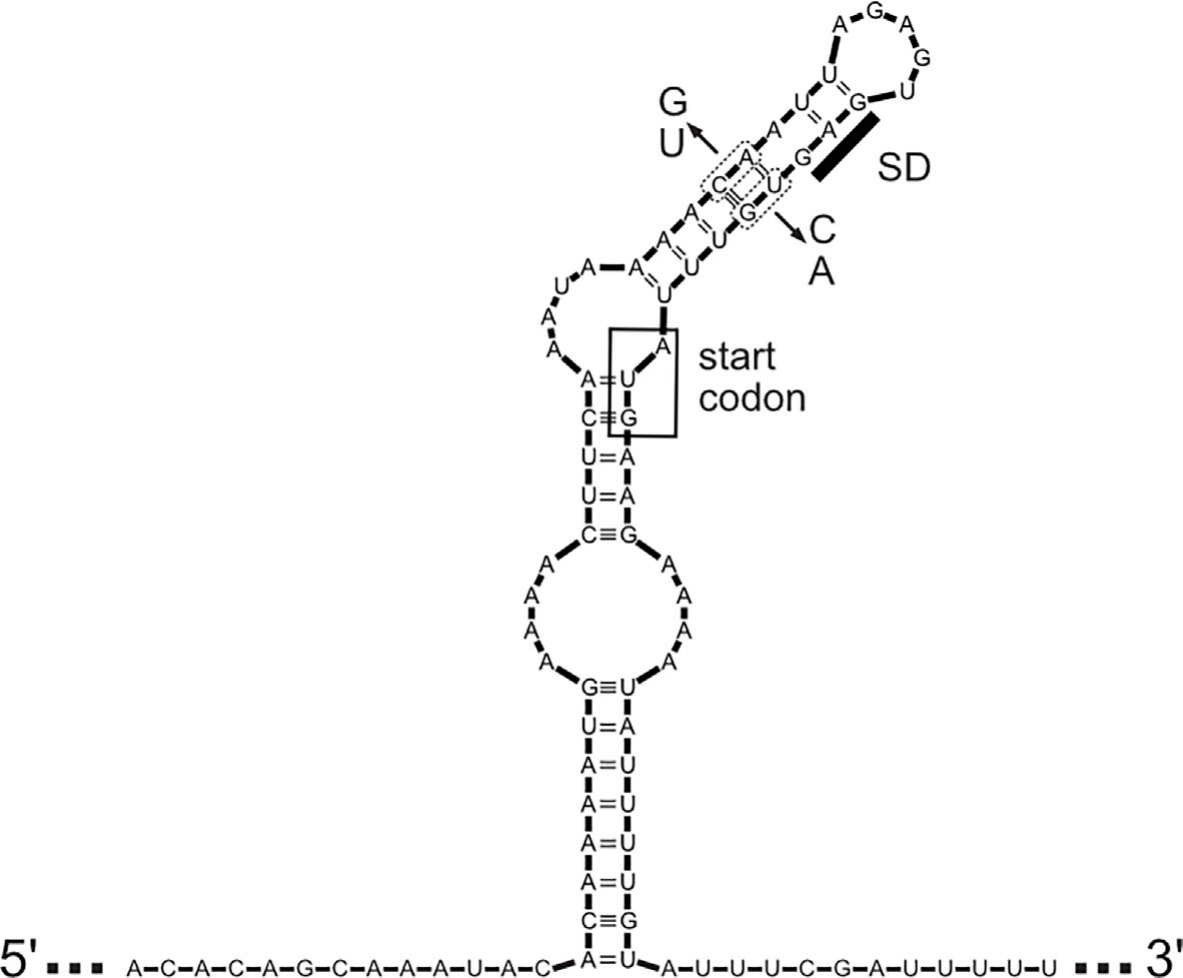
Predicted Structure of Leader Sequence. The predicted secondary structure of the mRNA leader sequence encompassing the coding sequences of *ligA* and *ligB* of *L. interrogans* sv Copenhageni str Fiocruz is shown. The Shine-Dalgarno sequence (SD) and AUG start codon (inside the box) are sequestered by the stem-loop structure. Location of mutations used to either disrupt or restore base pairing and test effects on expression are shown (see text for details).

There is signification post-transcriptional regulation of *lig* gene expression. **Figure 5** shows the predicted stem-loop structure that sequesters the ribosome-binding site and start codon of the *lig* genes (9). Deletion mutations that remove a portion of one strand of the stem increase ternary complex formation at the ribosome binding site *in vitro* and increase expression of a *lig’-’lacZ* translational fusion in *E. coli* (9). Mutations that disrupt base pairing in the stem also elevate expression of the fusion, and compensatory mutations that restore base pairing without restoring the wild-type sequence exhibit near wild-type levels of fusion expression. Elevation of the incubation temperature causes expression of the translational fusion to increase. These results support the existence of an “RNA thermometer” strategy for regulating *lig* gene expression, which has been identified in a number of bacterial pathogens, such as *Listeria monocytogenes* with lifestyles that include phases inside and outside of the mammalian host (40). Transcript levels of *ligA* and *ligB* were higher at 37°C than at 30°C (9) and higher during growth in a dialysis membrane chamber model in the rat compared to growth *in vitro* at 30°C (41). Transcript levels of *ligB* were also higher during the bacteremia of hamster infection (42). The simplest explanation for the effect of temperature on *lig* transcript levels is that the increased translation at the higher temperature protected the *lig* transcripts from degradation by RNases. Direct measurement of *lig* mRNA stability is required to test this hypothesis. It should be noted that the *ligB* gene is followed immediately by four small open reading frames, predicted to encode lipoproteins, that appear to be cotranscribed and coregulated with *ligB* in the same operon (11, 39).

The requirement of *ligB* for virulence was examined directly in two studies involving *L. interrogans*. A *ligB* mutant was generated in the Fiocruz L1-130 strain by targeted mutagenesis of *ligB* by replacing a segment of the gene with an antibiotic-resistance cassette (43). The mutant retained virulence by intraperitoneal inoculation into the hamster model of leptospirosis and caused histopathological damage. Although the *ligB* mutant also retained the ability to persistently colonize rats, a subtle effect of the mutation on renal colonization cannot be ruled out since the bacterial load in the kidneys was not quantified. Similarly, when hamsters were inoculated with a pool of leptospiral mutants carrying transposon insertions, the fitness of *ligB* mutant remained unaltered in blood, kidney, and liver (44). Because the hamsters were inoculated intraperitoneally in both studies, an effect of the *ligB* mutation on infection by a natural route of infection cannot be ruled out. Additionally, this study did not rule out the possibility that *ligA* was compensating for the loss of *ligB*.

A more recent study provided evidence that expression of either *ligA* or *ligB* is required for virulence (45). An artificial transcriptional repressor of the *lig* genes was constructed by using a Transcription Activator-Like Effector (TALE) and inserted into a transposon harbored on a suicide plasmid. The plasmid was transferred to the L495 strain of *L. interrogans* by conjugation, and the transposon was allowed to randomly insert into the chromosome. LigA and LigB levels were diminished in three transconjugants, two of which were avirulent following intraperitoneal inoculation into hamsters. Blood and kidneys were culture negative at death, indicating that expression of either *ligA* or *ligB* was required not only for virulence but also for renal colonization. These results support the conclusion that LigA and LigB have overlapping essential functions in virulence. It is important to note that the *ligC* gene is intact in *L. interrogans* serovar Manilae, strain L495, suggesting a distinct, non-redundant function for LigC. A *ligC* transposon insertion mutant in the strain L495 retained virulence (46).

## Interactions With the Host Extracellular Matrix

The extracellular matrix (ECM) is a complex mixture of fibrous proteins, proteoglycans, and glycoproteins that are found in the spaces between cells and provide for their support. Together with cell adhesion proteins, they are the glue that holds tissues together. They also provide tracks along which cells migrate during development, immune response, tissue repair, and other processes requiring cell movement.

Some of the earliest studies of leptospiral attachment found that *L. interrogans* adhered to the ECM and to the individual ECM components fibronectin, type I collagen, and laminin and that the level of adherence was correlated with leptospiral virulence (47, 48), a characteristic correlated with Lig protein expression (13). Over 40 leptospiral proteins have been identified that bind to selected extracellular matrix components (49, 50). For most of these putative ECM adhesins, evidence comes primarily from *in vitro* studies that examine interactions between purified recombinant proteins and ECM components. The biological significance of some of these interactions has been questioned (51) because it seems unlikely that such a large number of adhesins would have redundant roles in the interaction with the extracellular matrix *in vivo*.

The designation of LigA and LigB as ECM binding proteins is based on a range of evidence. The binding of LigA and LigB to purified fibronectin and other components of the ECM has been examined *in vitro* with purified recombinant Lig proteins. Because full-length Lig proteins are difficult to express in *E. coli*, shorter fragments of LigA and LigB that collectively spanned both proteins were used in these studies. High-affinity binding to fibronectin was observed with a fragment harboring the carboxyterminal Ig-like domains of LigA (LigA7’-13, **Figure 1**) and LigB (LigB7’-12, LigBCen, **Figure 1**) (32, 52, 53). Binding affinity was highest when the first Big_5 domain was included with LigB7’-12 (LigB-U, **Figure 1**) (52). The N-terminal Ig-like domains common to LigA and LigB did not bind fibronectin (52, 54). A weak binding site with a dissociation constant in the micromolar range was also detected in the C-terminal half of the CTE of LigB in two independent studies (LigBcte’ and LigBctv, **Figure 1**) (52, 54). The binding site in the CTE was narrowed down to an 11 amino acid peptide containing a critical Leu-Ile-Pro-Ala-Asp sequence, which attached to the 15th type III module of fibronectin (55). Differential binding activities for extracellular matrix proteins were observed for LigA and LigB. The affinity of LigA7’-13 for fibronectin was weaker than that of LigB7’-12 (apparent K_d_ of 126 nM vs. 73 nM) (52), although similar affinities of LigA7’-13 and LigB7’-12 for the gelatin-binding domain of fibronectin were observed by another group (53). LigB7’-12 also attached with moderate affinity to collagen I, collagen IV, and laminin, whereas binding of LigA7’-13 to the collagen molecules was poor and to laminin undetectable (52). Finally, LigBCon and LigBCen had moderate affinity for lung elastin, whereas LigBctv adherence to elastin was undetectable (56). Adherence of LigA7’-13 to elastin was not examined. In gain-of-function studies with the poorly adhesive *L. biflexa*, heterologous expression of LigA and LigB on the surface of this organism significantly enhanced its ability to adhere to fibronectin and laminin (57). However, production of LigA or LigB did not increase attachment of *L. biflexa* to elastin, collagen I, or collagen IV, despite the host molecules being bound with moderate affinity by recombinant LigB.

*L. interrogans* adheres to a variety of cultured mammalian cells, including MDCK canine kidney cells (58, 59). Adhesion to MDCK cells is inhibited if *L. interrogans* is premixed with fibronectin prior to addition of leptospires to the monolayer, suggesting that adherence of *L. interrogans* to MDCK cells relies in part on fibronectin (54). LigBCen and LigBctv (**Figure 1**) attached to MDCK cells in a dose-dependent manner, although binding of LigBctv was weaker. This result is consistent with the stronger affinity of LigBCen over LigBctv for fibronectin. Binding of both LigBCen and LigBctv were reduced in MDCK cells in which fibronectin expression was knocked down with siRNA, further supporting the notion that fibronectin serves a receptor for LigB-mediated adhesion of *L. interrogans* to MDCK cells. Moreover, LigBCen and LigBctv inhibited adherence of *L. interrogans* when the MDCK cells were pretreated with the proteins (54). However, an *L. interrogans ligB* mutant adhered to MDCK cells as well as the wild-type strain (43). The discrepancy between the lack of effect of the *ligB* mutation on adherence and the inhibitory activity of LigB protein segments on adherence may be explained by the presence of additional adhesins that target the same host protein. It is likely that the LigB proteins prebound to MDCK cells block other fibronectin-binding adhesins expressed by *L. interrogans*, such as LigA. In general, interpretation of experiments whose goal is to assign adhesin activity to a microbial surface protein by deploying it an inhibitor are complicated by the presence of additional surface proteins targeting the same host receptor. In this case, gain-of-function experiments with *L. biflexa* expressing the Lig proteins are helpful. In fact, *L. biflexa* producing LigA adhered to MDCK cells better than *L. biflexa* alone (7). Surprisingly, surface expression of LigB did not enhance adherence of *L. biflexa* to MDCK cells. This result is possibly due to proteolysis of LigB in *L. biflexa* leaving behind a slightly smaller version of LigB as its major species, as revealed in Western blots (7). The lost segment of LigB may have contained the fibronectin-binding sequence in LigBctv (54).

Fibronectin-binding proteins mediate bacterial adhesion to and invasion of host cells (60). For example, binding of fibronection by *Staphylococcus aureus* fibronectin-binding proteins enables over a 100-fold increase of intracellular bacteria within epithelial and endothelial cells (61, 62). Similarly, *L. biflexa* expressing LigB is taken up by mouse macrophages and exhibits enhanced binding to fibronectin compared with *L. biflexa* lacking LigB (38). Virulent *L. interrogans* invades the murine monocyte-macrophage-like cell line J774A.1 and induces apoptosis (63). A small population of leptospires that escaped killing also multiplied in a vacuole of mouse macrophages (64). *L. interrogans* injected into zebrafish embryos also invade macrophages, which subsequently traffic to the kidneys (65). However, a *ligB* mutant was able to infect mouse macrophages as well as wild-type *L. interrogans*, whereas an *lmb216* mutant was significantly impaired in invading macrophages (38), which calls into question whether LigB is significantly involved in macrophage invasion.

## Evasion of Innate Immunity Including Complement

Leptospires must fight off a number of components of host innate immune system to establish an infection. During the initial encounter with the host, the spirochetes immediately encounter physical barriers, antimicrobial peptides, and other elements of the innate immune system. They must also deal with the cellular elements of innate immunity, including neutrophils and macrophages. During dissemination in the bloodstream, leptospires must evade the bactericidal activity of complement. Leptospires that colonize the proximal tubular lumen of the kidney also encounter complement due to local production by the kidney (66, 67). The three complement pathways, classical, lectin-binding, and alternative, are triggered by different processes that converge on the cleavage of C3 into C3a and C3b near the surface of microbes. The covalent binding of C3b to the microbial surface leads to direct killing of the target microbe through assembly of C5 convertase and assembly of the lethal membrane attack complex. Indirect killing occurs because C3b is an opsonin that is recognized by specific receptors on phagocytes, which are attracted by the anaphylatoxins C3a and C5a released by cleavage of C3 and C5 by the respective convertases.

Sensitivity of *Leptospira* species to serum killing is related to their pathogenicity. The noninvasive saprophyte *Leptospira biflexa* is rapidly killed in 40% normal human serum (NHS) within 5 min (68). On the other hand, pathogenic leptospiral strains exhibit various degrees of resistance to serum killing (69). Serum resistance of pathogenic leptospires is likely due to complement resistance mechanisms. Additional studies are needed to determine which complement pathways are responsible for controlling *L. interrogans* infections. The survival of one *L. interrogans* strain with intermediate levels of resistance to normal human serum was enhanced in serum deficient for the complement component C1s, suggesting that serum resistant strains of *L. interrogans* are equipped to resist the classical complement pathway (69). Human sera that test negative for *Leptospira*-specific antibodies with the microscopic agglutination test contain IgG that binds to *Leptospira* strains (69, 70). Therefore, the classical complement pathway may be triggered on the surface of *L. interrogans* by nonspecific IgG in individuals never exposed to *Leptospira* previously.

Pathogenic leptospires incubated in normal human serum capture the complement regulators factor H, FHL-1, FHR-1, and C4b-binding protein (C4BP) (68, 69). The principal role of these complement regulators is to prevent complement activation on host-cell surfaces by acting as a cofactor for the protease factor I, which cleaves C3b and C4b, components of the alternative and classical pathways, respectively. They also accelerate disassembly of the convertases. Except for FHL-1, the complement regulators captured by *L. interrogans* retained cofactor activity for factor I, which cleaves C3b and C4b. In contrast, *L. biflexa* bound negligible amounts of factor H and C4BP. To examine the role of the Lig proteins in the capture of complement regulators, separate plasmids carrying the *ligA* and *ligB* genes were transformed into *L. biflexa*. Heterologous production of LigA or LigB on the surface of *L. biflexa* resulted in enhanced survival in normal human serum (8, 71). The transformed strains captured factor H and C4BP from human serum and cleaved C3b and C4b when added along with the substrates to the Lig-producing *L. biflexa* pre-incubated with human serum or purified complement regulators (8). Membrane attack complex (MAC) deposition on the surface was also reduced in the transformants, although it remained greater than the level of MAC on *L. interrogans*. Recombinant LigA7'-13 and LigB7'-12 also bound to factor H and C4BP, which retained the cofactor activities leading to proteolysis of C3b and C4b, respectively. Although LigB0-7’ was also shown to bind both complement regulators at room temperature (72), a later study showed that attachment of LigB0-7’ to C4BP during incubation at physiologic temperature was barely detectable (73). The Lig proteins also attached to factor H related-1 (FHR-1) and factor H like-1 (FHL-1) proteins (72). These results suggest that *L. interrogans* uses LigA and LigB to capture host complement regulators to defend itself against complement.

Additional studies provided direct evidence that LigB inhibits complement pathways. A segment of LigB that hinders complement activity was identified with complement-dependent hemolytic assays (71). LigB9-11 that was preincubated with human serum inhibited lysis of antibody-sensitized sheep erythrocytes by human complement, indicating that LigB inhibits the classical complement pathway. C4BP binds with high affinity to LigB9-10 (73), indicating that C4BP is at least partially responsible for LigB-mediated resistance to the classical pathway. Additional high-affinity sites for C4BP were mapped to LigA7-8 and LigA10-11. Similarly, LigB9-11 preincubated with human serum suppressed lysis of rabbit erythrocytes by human complement, indicating that LigB inhibited the alternative complement pathway as well, presumably by acquiring factor H from the host.

Curiously, LigB9-11 also binds with high affinity to C3b and C4b (71). The reason for LigB capturing such lethal host proteins is unknown. However, the outer surface protein OspC of Lyme *Borrelia* blocks formation of C3 convertase by competing with complement factor C2 for binding to C4b (74). Similarly, Efb of *Staphylococcus aureus* binding to C3b allosterically inhibited its binding to complement factor Bb (75).

The CCP (complement control protein) modules of the complement regulators bound by the Lig proteins were identified using a panel of C4BP mutants lacking single CCP domains in its α chain (73). Deletion of CCP1, CCP2, or CCP3, which are involved in binding C4b, has no effect on binding of LigA7’-13 or LigB7’-12. Instead, removal of CCP7 or CCP8 diminished binding of both Lig proteins (73). Several other bacterial pathogens also interact with C4BP *via* CCP7 and/or CCP8. LigA7’-13, LigB7’-12, and LigB0-7’ also binds to a fragment of factor H encompassing the six C-terminal CCP domains, CCP15-20 (72). More importantly, two different monoclonal antibodies raised against CCP20 inhibited binding of LigB7’-12 by 50% suggesting that LigB7’-12 targets CCP20 for binding. CCP19-20 is critical for factor H function because CCP20 harbors the site for binding to host cells and CCP19 binds to C3b. A variety of microbes use the same site in CCP20 to bind factor H, including the spirochetes *Borrelia burgdorferi* and *Borrelia hermsii* (76–79). It is possible that *L. interrogans* uses LigB to interact with factor H at the same site in CCP20.

Additional complement resistance mechanisms involve capturing plasminogen and secreting metalloproteases that cleave complement components (80). Plasmin is a tightly controlled protease that plays a central role in the fibrinolytic system by dissolving fibrin clots after wound repair. Plasmin is generated from cleavage of the proenzyme plasminogen by one of several proteases, including urokinase-type plasminogen activator (uPA). *L. interrogans* exploits plasmin to cleave and inactivate C3b and IgG bound to its surface (81). *L. interrogans* captures plasminogen, which is converted by exogenously added urokinase-type plasminogen activator (uPA) into plasmin (82). The LigB7’-12 fragment binds with high affinity to plasminogen (K_D_ = 95 nM). LigA7’-13 also binds plasminogen, but with much lower affinity (K_D_ = 1.2 µM) (70). When plasmin is generated from the bound plasminogen by the addition of uPA, not only does plasmin cleave its physiologic substrate, fibrinogen, but it also cleaves C3b, and C5 *in vitro*. These results suggest that LigA and LigB help *L. interrogans* resist complement not only by acquiring host complement regulators but also by proteolysis of complement components with a captured host protease.

## Modulation of Hemostasis

Hemorrhage is a well-known hallmark of leptospirosis, with a majority of patients manifesting mild bleeding of the skin and mucous membranes to severe pulmonary hemorrhage and gastrointestinal bleeding (83). In a study of 52 consecutive patients with severe leptospirosis in Semarang, Indonesia, 60% of patients had clinical evidence of bleeding (84). At autopsy, widespread hemorrhaging has been described at mucosal surfaces and various organs including the heart, lungs and kidneys (85). Laboratory measures of coagulation (e.g., prothrombin time) and fibrinolysis (e.g., D-dimer levels) are typically abnormal in leptospirosis (83, 84). These changes in hemostatic pathways could be due to either a sepsis-like host response to leptospirosis (i.e., disseminated intravascular coagulation), direct effects of *L. interrogans* (86–89), or both.

**Figure 6:**
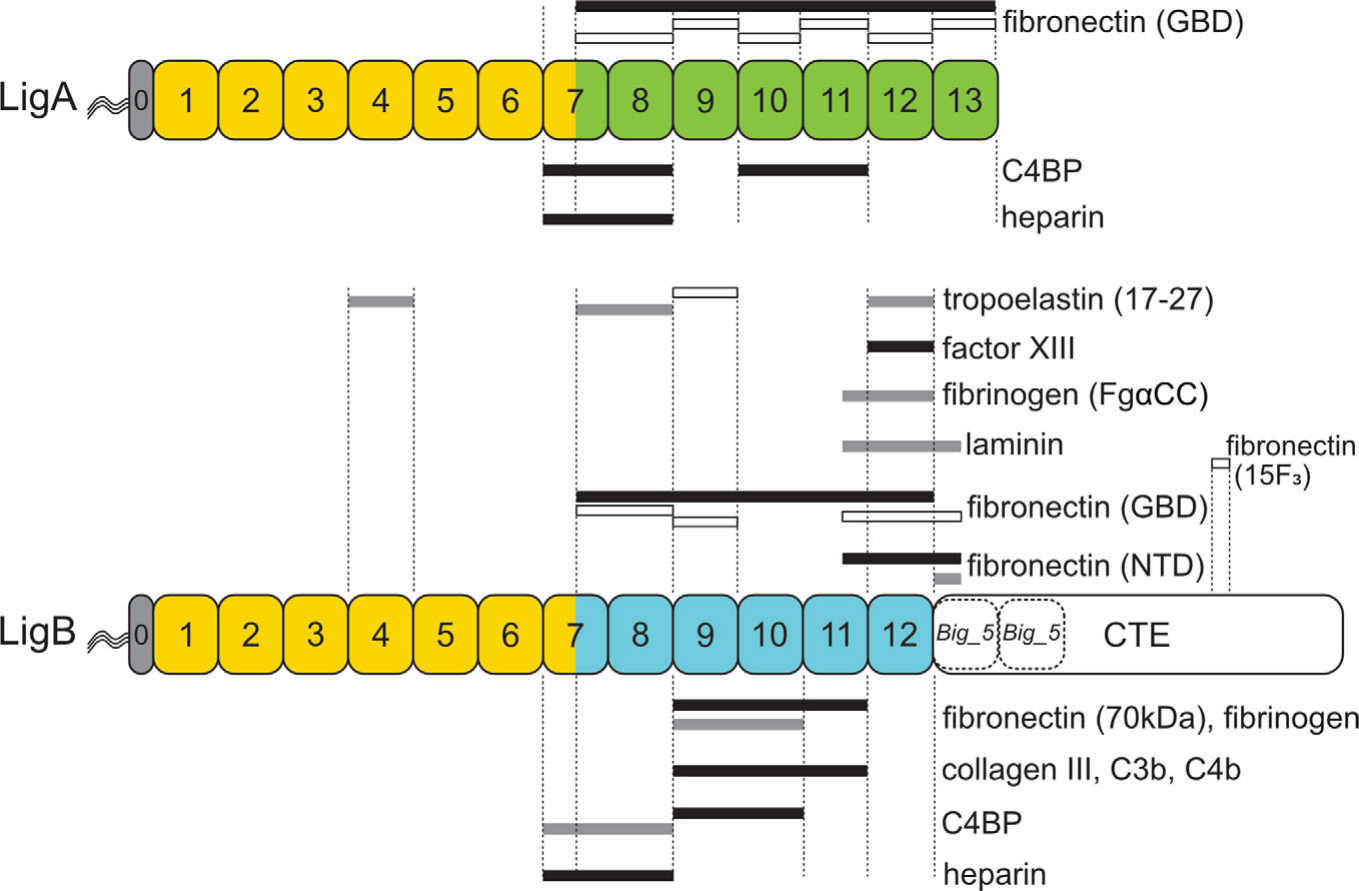
Leptospiral Immunoglobulin-Like B (LigB) Domains Involved in Interactions with Host Proteins. Regions of LigA and LigB involved in interactions with host proteins are shown for heparin (73), C4 binding protein (C4BP) (73), tropoelastin (56), factor XIII (91), fibrinogen (6, 92), laminin (90), collagen III (6), complement components C3b and C4b (71), the gelatin-binding domain (GBD) of fibronectin (53), the amino-terminal domain (NTD) of fibronectin (32, 93), the 70 kDa fragment of fibronectin (6), and the 15th type III module of fibronectin (15F3) (55). The color scheme used for the Lig proteins is identical to that of **Figure 1**. Nearly identical sequences between LigA and LigB are shown in yellow. The remaining Big_2 domains are shown in green (LigA) and blue (LigB). The C-terminal extensions (CTE) contain putative Big_5 domains (dashed). Black bars indicate high-affinity binding (< 100 nM) to the host target, gray bars indicate moderate affinity (100–800 nM), and open bars indicate low affinity (> 1 µM).

Impaired coagulation and enhanced fibrinolysis are likely to facilitate leptospiral invasion and dissemination in the case of a mucosal or skin wound as the site of initial infection. Local impairment of hemostasis could act as a form of immune evasion by preventing entrapment in fibrin strands. There is *in vitro* evidence that the Lig proteins could be playing one or more roles in these processes through their interactions with host coagulation factors and platelets. Both LigA and LigB bind to fibrinogen as purified proteins (52, 90) and increased fibrinogen binding when expressed on the surface of *L. biflexa* (6). The region of LigB responsible for binding was identified to be domains 9-11 (**Figure 6**), which was found to have high affinity for fibrinogen and fibrinogen fragment D, with K_D_s of ~28 nM (6). The interaction with fibrinogen fragment D is important because fragment D is responsible for cross-linking fibrin monomers during coagulation. The affinity of LigB domains 9-11 (LigB9-11) for fragment D may be responsible for the ability of LigB9-11 to block thrombin-catalyzed fibrin clot formation (6).

LigB9-11 overlaps with LigBCen2R, a region of LigB comprising a portion of domain 11 and all of domain 12. LigBCen2R bound to the C-terminal αC (FgαCC) domain of fibrinogen, a component of fragment D (92). Like LigB9-11, LigBCen2R inhibited fibrin clot formation, and also blocked platelet adhesion and aggregation (92). Interestingly, a region of LigB similar to LigBCen2 was enriched from among 280,000 phage clones in a screen for heparin binding proteins (94). Another group mapped additional heparin-binding sites to LigB7-8 and LigA7-8 (73). This is relevant to hemostasis because heparin is a host glycosaminoglycan that inhibits clot formation. Subsequently, LigB domain 12 (LigB12) was found to interact not only with the FgαCC domain of fibrinogen but also with factor XIII, which acts to cross-link fibrin to form a more stable clot (91). LigB12 blocked binding of factor XIII to FgαCC and prevented factor XIII-mediated cross-linking of fibrinogen. This discovery suggests a second possible mechanism for LigB to inhibit coagulation, and this study found that like LigB9-11 and LigBCen2R, LigB12 was able to block thrombin-catalyzed fibrin clot formation (91).

Fibrinolysis involves digestion of fibrin clots, primarily by the proteolytic activity of plasmin. Plasminogen has been shown to bind to the leptospiral surface and become activated to plasmin (82). As discussed earlier, the Lig proteins may be responsible for some of the binding of plasminogen to the leptospiral surface. Both LigA and LigB bind plasminogen, and Lig-bound plasminogen can be converted to active plasmin in the presence of urokinase-type plasminogen activator (8). Thus, expression of the Lig proteins on the leptospiral surface provides a way for leptospires to cleave fibrin as a dissemination and/or immune evasion mechanism.

## Characterization of Domains Involved in Host Protein Interactions

Deletion studies *in vitro* with LigB by several groups have narrowed down the contact points of LigB with most host molecules examined to discrete segments within domains 9 through 11 (**Figure 6**) (6, 71, 73). The 70 kDa N-terminal domain of fibronectin, fibrinogen, collagen III, C3b, and C4b make high-affinity contact with LigB9-11, and C4BP binds tightly to LigB9-10 (**Figure 6**). At least for fibronectin and fibrinogen, LigB domain 11 contributes less strongly to the interactions. The evidence for this is that LigB9-10 retained moderate affinity for fibronectin and fibrinogen (~150-250 nM), whereas binding to the host proteins by LigB10-11 was not detected (6). On the other hand, domains 9 and 10 are critical for binding activity. In contrast to the effect of removing domain 11 from Lig9-11, removal of the amino-terminal half of domain 9 or even placing a histidine tag (His6) at the amino terminus of LigB9-11 eliminated binding. Removal of domain 10 from LigB7’-10 resulted in weak binding to fibronectin and fibrinogen (6). From these observations, the authors proposed that high-affinity contacts occur between LigB and fibronectin and fibrinogen within domains 9 through 10, with domain 11 serving to enhance binding by affecting the conformation or positioning of the other domains (6). The kinks detected in the LigB structure may be important in interdomain interactions that lead to high-affinity binding with host proteins (29).

Another group has demonstrated through a series of studies that the binding of LigB to fibronectin, fibrinogen, and factor XIII centers on domain 12 (54, 91–93). Although the involvement of LigB12 in binding to fibronectin seems to contradict the findings by Choy et al. at first glance (6), Lin *et al*. found that the high-affinity binding of LigB12 to the NTD of fibronectin required an additional 47 residues beyond domain 12, extending into the first putative Big_5 domain in the CTE (93). LigB12 missing the additional sequence failed to bind the NTD of fibronectin, which is consistent with the lack of binding to fibronectin observed by Choy et al. with LigB11-12. On the other hand, the observed moderate affinity of LigBCen2R (part of domain 11 and all of domain 12) for fibrinogen (92) is contradicted by the complete lack of binding observed with LigB11-12 (6). A possible reason for the discrepancy is that the 6-histidine tag was placed at the carboxy terminus of LigB11-12 and at the amino terminus of LigBCen2R (6, 92).

Fibronectin binding requires conformational flexibility of the Lig proteins. High-affinity binding of LigBCen2 to the NTD of fibronectin requires Ca^2+^ (32). The far-UV CD spectrum of LigB Cen2 in the absence of calcium suggests that although the protein is largely a β-sheet structure, the protein is partial unfolded (32). In the presence of calcium, LigBCen2 gains β-sheet structure. The key segment in LigBCen2 for interaction with the NTD of fibronectin lies within the 47-residue segment beyond the C-terminus of LigB12 rather than within LigB12. In the presence of calcium, binding to the NTD by LigBCen2R, which lacks the 47 residues, was not detected (93). The 47 amino acid segment alone, LigBCen2NR, which comprises the initial portion of the first Big_5 domain in the CTE, retained moderate binding affinity for the NTD. Several biophysical experiments indicated that LigBCen2NR was highly disordered and extended. The β-sheet conformation of LigBCen2NR increased considerably upon addition of the NTD of fibronectin, akin to the β-zipper mode of binding described for other fibronectin-binding proteins (95). On the other hand, the NTD did not undergo major conformational changes upon binding LigBCen2NR (93). Further studies are needed to understand the role of calcium in the conformation of LigBCen2NR in relation to the 47-residue extension.

Binding of LigA and LigB may involve multiple domains contacting host proteins in an avidity effect. LigA7’-13 and LigB7’-12 bind with high affinity to the GBD of fibronectin, yet none of the individual domains were able to attach with even moderate affinity (53). Weak binding was detected with domains LigB7’-8, LigB9, and LigBCen2 (**Figure 6**). Binding was not measurable with LigB10 or LigB11 (53). Weak binding to the GBD was also detected with LigA7’-8 and the individual LigA domains from LigA9 through LigA13. These results suggest that LigA and LigB binding to GBD involves multidomain interactions. In another study, *L. interrogans* was shown to adhere to elastin and tropoelastin. LigBCon (LigB1-7’) and LigBCen (LigB7’-12 plus half the C-terminal domain) had moderate affinity for tropoelastin, while binding by LigA7’-13 was not detected (56). Deletion studies narrowed down the binding sites for tropoelastin to LigB4 and LigB12, but weaker binding sites were also detected with LigB7’-8 and LigB9 (**Figure 6**). Thus, interaction of the Lig proteins with fibronectin and tropoelastin may involve multiple Ig-like domains making simultaneous contact with the host proteins.

LigB4 was selected for further analysis since it had the highest binding affinity for tropoelastin. The affinity of LigB4 for tropoelastin was affected by pH changes, although the secondary structure was unaffected. This indicated that an ionic interaction was involved in the LigB-tropoelastin interaction. Since the tropoelastin fragment used for the experiment was positively charged, the sole acidic amino acid conserved in LigB4, LigB7’-8, LigB9, and LigB12 was changed to asparagine in LigB4. This D341N mutation caused reduced affinity of LigB4 for tropoelastin without affecting its secondary structure (56). In the NMR structure of LigB12, the asparate residue corresponding to position 341 is located in the loop between strands B and C at the external edge of sheet 1a, where its side chain is well positioned to interact with a host macromolecule (**Figures 2** and **3A**).

Amino acid residues in LigB12 important for binding to FgαCC8 were also identified by targeted mutagenesis (91). The D1061A, F1054A, and A1065K mutations decreased binding of LigB12 to FgαCC8. None of the three changes affected the secondary structure of LigB12. All three changes are located in sheet 1a, which extends out from the rest of the structure of LigB12 (**Figure 3A**). Although the aspartate at position 1061 is conserved among the LigB Ig-like domains, the phenylalanine at position 1054 and alanine at 1065 are not present in the other LigB domains, which do not bind FgαCC8 (**Figure 2**). Thus, LigB uses sheet 1a of its Ig-fold to interact with tropoelastin and fibrinogen (56, 91).

## Serodiagnosis

LigA and LigB are potential serodiagnostic antigens. As mentioned above, the *lig* genes were discovered by probing expression libraries with convalescent sera. LigB seroreactivity is present in 81% of Brazilian patients with leptospirosis for less than 7 days of illness, including many patients who had not yet formed antibodies detectable by whole cell IgM enzyme-linked immunosorbent assay (ELISA) and microagglutionation test (MAT) (96). LigA seroreactivity has also been shown to be present in high percentages of patients with acute leptospirosis in Thailand an average of 7 days after onset of fever (97) and in the Philippines at the time of hospital admission (98). Lig antigens have also been found to be useful serodiagnostic antigens for canine (99) and equine (100, 101) leptospirosis. In a proteome microarray screen comparing K_D_ 2,241 leptospiral proteins (61% of the *L. interrogans* proteome), LigA7-13 and LigB7-12 had the highest IgG area under the curve (AUC) among all proteins studied for reactivity with sera obtained from patients with acute leptospirosis (at the time of admittance to the hospital) compared to sera from healthy controls in an area of high endemicity for leptospirosis (102). A follow-up proteome microarray study examining reactivity of sera from patients with mild and severe disease (requiring hospitalization) found that the domains shared by LigA and LigB (LigA/B1-6) had the highest accuracy for diagnosis of both forms of infection (103). The high levels of Lig seroreactivity in patients with acute leptospirosis suggests that the Lig proteins are expressed early in infection.

## Vaccine Studies

The Lig proteins are among the most promising subunit vaccine candidates for immunoprotection against leptospirosis. At least 21 studies have examined immunoprotection with either recombinant Lig proteins or *lig* DNA (**Table 1**). The seminal Lig vaccine study, published in 2004 by Koizumi and Watanabe, provided evidence for immunoprotection from lethality (but not renal colonization) with both LigA and LigB expressed as recombinant proteins in which all of the Ig-like domains were included (14). Unlike most vaccine studies, which involve hamsters, the animal model was 4-week-old C3H/HeJ mice. Hamsters are highly susceptible to overwhelming, disseminated infection, while mice are considerably less susceptible though they may develop renal colonization. C3H/HeJ mice have a defect in TLR4, which impairs their innate immune response to leptospiral lipopolysaccharide (LPS) and increases their susceptibility to leptospiral infection. In addition, this study involved a mouse-adapted *L. interrogans* serovar Manilae strain that was inoculated by intraperitoneal inoculation at a challenge dose of 10^6^ organisms, 1000-times greater than the ED_50_ dose.

**Table 1:** Lig Vaccine Studies.

Publication	Immunization				Challenge				Survival**		*P* value	
1st Author, Year	Antigen Type	Region*	Adjuvant	Immunization Route	Model	Route	Dose	ED_50_	Control	Test	Fisher's Exact	Log-Rank
Koizumi et al., 2004 (14)	His6 RPS-*Ec*	LigA d1'-d13 (68-1224)	Freund's	SQ	Mouse C3H/HeJ	ip	10^6^	<10^3^	2/5	9/10	NS	0.025
		LigB d1'-d12 (68-1191)								9/10	NS	0.036
Palaniappan et al., 2006 (12)	GST RPS-*Ec*	LigA d0'-d7' (32-630) + LigA d7'-d13 (631-1225)	Al(OH)_3_	SQ	Hamster	ip	10^8^	10^8^	7/8	8/8	NS	NA
									4/5	5/5	NS	NA
									4/7	8/8	NS	NA
Silva et al., 2007 (104)	His6 RPF-*Ec*	LigA d7'-d13 (625-1224)	Freund's	SQ	Hamster	ip	10^3^	45	0/10	8/10	<0.001	<0.0001
		LigB d2-d7' (131-645)								0/10	NS	NS
		LigB d7'-d12 (625-1259)								1/10	NS	NS
Faisal et al., 2008 (105)	DNA	LigA d0'-d7' (32-630) + LigA d7'-d13 (631-1225)	none	IM	Hamster	ip	10^8^	10^8^	4/8	8/8	NS	NA
									5/8	8/8	NS	NA
									6/8	8/8	NS	NA
Faisal et al., 2009 (106)	RPS-*Ec*	LigA d7'-d13 (631-1225)	Al(OH)_3_		Hamster	ip	10^8^	10^8^	0/8	4/8	NS	<0.01
			liposome	SQ					0/8	7/8	<0.01	<0.001
			PLGA						0/8	6/8	<0.01	0.001
Yan et al., 2009 (107)	RPS-*Ec* + His6 RPS-*Ec* + RPS-*Ec*	LigB d0'-d7' (32-630) + LigB d7'-d12 (631-1418) + LigB CTE (1419-1890)	Al(OH)_3_	SQ	Hamster	ip	2×10^8^	10^8^	1/8	7/8	0.01	0.001
	RPS-*Ec*	LigB d0'-d7' (32-630)								6/8	<0.05	<0.01
	His6 RPS-*Ec*	Lig B d7'-d12 (631-1418)								4/8	NS	NS
	RPS-*Ec*	LigB CTE (1419-1890)								3/8	NS	NS
Cao et al., 2011 (108)	RPS-*Ec*	LigB d0'-d7' (32-630)	Emulsigen	SQ	Hamster	ip	250	100	0/6	2/6	NS	<0.05
		LigB d4 / LigB d12								3/6	NS	<0.02
		LigB d4-d7' / LigB d12								3/6	NS	<0.05
Coutinho et al., 2011 (109)	His6 RPS-*Ec*	LigA d7'-d13 (631-1224)	Freund's	SQ	Hamster	ip	1000	50	0/4	4/4	<0.05	0.01
		LigA d7'-d11 (631-1033)								2/4	NS	0.01
		LigA d7'-d9 (631-851)								0/4	NS	NS
		LigA d10-d13 (852-1224)								4/4	<0.05	0.008
		LigA d10-d12 (852-1124)								4/4	<0.05	0.008
		LigA d11-d13 (943-1224)								4/4	<0.05	0.008
		LigA d11-d12 (943-1124)								1/4	NS	NS
		LigA d12-d13 (1034-1224)								2/4	NS	0.01
Lucas et al., 2011 (110)	His6-RPI-*Ec*	LigA d7'-d13 (625-1224)	Al(OH)_3_	NA	Hamster	ip	2000	10	2/10	0/10	NS	NA
Forster et al., 2013 (111)	DNA	LigA d7'-d13 (629-1224)	Al(OH)_3_	IM	Hamster	ip^h^	10	2	0/6	0/8	NS	NS
		LigB: d7'-d12 (629-1112)								0/8	NS	NS
		LigB d0-d7' (1-628)								5/8	<0.05	<0.01
		LigB CTE (1113-1501)								0/8	NS	NS
		LigB CTE (1502-1891)								0/8	NS	NS
Lourdault et al., 2014 (112)	Lipoprotein	OspA SP / LigA d7'-d13 (631-1224)	*E. coli*	oral	Hamster	ip	1000	20	0/8	3/8	NS	<0.0001
						id	100	<10	0/8	5/8	<0.05	<0.02
Hartwig et al., 2014 (113)	His6 mRP-*Pp*	LigA d7'-d13 (625-1224)	Al(OH)_3_	IM	Hamster	ip	1300	36	0/6	6/6	<0.01	<0.01
	His6 dmRP-*Pp*	LigA d7'-d13 (625-1224)								0/6	NS	NS
Bacelo et al., 2014 (114)	His6 RPF-*Ec*	LigA d7'-d13 (625-1224)	Al(OH)_3_	SQ	Hamster	ip	1300	36	0/6	4/6	NS	<0.001
			CpG						0/6	0/6	NS	NS
			xanthan						1/5	6/6	<0.05	<0.01
			CpG + xanthan							6/6	<0.05	<0.01
Forster et al., 2015 (115)	RPF-*Ec* ^p,b^	LigB d0-d7' (1-628)	Al(OH)_3_	IM	Hamster	ip^h^	10	2	0/5	0/6	NS	<0.05
	DNA^p^/RPF-Ec^b^									5/6	<0.02	<0.01
	DNA^p^/DNA^b^									2/5	NS	<0.01
	DNA^p^/DNA^b^		none							0/5	NS	NS
Oliviera et al., 2016 (116)	His6 RPF-*Ec*	LigA d7'-d13 (625-1224)	Al(OH)_3_	SQ	Hamster	ip	1300	260	0/6	4/6	NS	<0.001
			CpG						0/6	1/6	NS	<0.01
			CpG + MWCNT						0/6	1/6	NS	<0.01
			MWCNT						0/6	0/6	NS	<0.01
Conrad et al., 2017 (117)	His6 RPF-*Ec*	LigB d2-d7' (131-645)	Al(OH)_3_	IM	Hamster	ip	200	18	0/10	10/10	<0.0001	<0.0001
Evangelista et al., 2017 (118)	His6 RPS-*Ec*	LigA d7'-d13 (631-1224)	Freund's	SQ	Hamster	ip	10^5^	20	0/5	8/8	<0.001	0.0001
		LigB d0-d7 (19-672)								3/8	NS	NS
		LigA d7'-d11 (631-1224) + LigB d0-d7 (19-672)								8/8	<0.001	0.0001
Hsieh et al., 2017 (31)	(NA) RPS-*Ec*	LigB d7 (579-674)	Al(OH)_3_	SQ	Hamster	ip	250	NA	1/6	0/6	NS	NS
		LigB d10 (848-943)								0/6	NS	NS
		LigB d10'(848-889) / d7'(621-674)								6/6	<0.02	<0.01
da Cunha et al., 2019 (119)	His6 RPF-*Ec* ^p,b^	LigA d11-d13 (943-1224) / LigB d2-d7' (131-648)	Al(OH)_3_	IM	Hamster	ip	200	40	0/8	8/8	<0.001	<0.001
	His6 RPF-*Ec* ^p,b^		Montanide							8/8	<0.001	<0.001
	DNA^p^/DNA^b^		none/none							2/8	NS	NS
	DNA^p^/H6-RPF^b^		none/ Al(OH)_3_							8/8	<0.001	<0.001
Techawiwattanaboon et al., 2019 (120)	His6 RPF-*Ec*	LigA d7'-13 (631-1224)	liposome + MPL + QS21	SQ	Hamster	ip	200	20	0/5	3/5	NS	<0.05
		LigA d7'-d13 (631-1224) + LenA + LcpA + Lsa23								3/5	NS	<0.01
Techawiwattanaboon et al., 2020 (121)	His6 RPF-*Ec*	LigA d7'-13 (631-1224)	liposome + MPL + QS21	IM	Hamster	ip	200	20	0/5	3/5	NS	<0.05
				SQ						3/5	NS	<0.05

Al(OH)_3_, aluminum hydroxide; GST, glutathione-S-transferase; RPS-Ec, recombinant protein produced in E. coli and purified as soluble protein; RPF-Ec, recombinant protein produced in E. coli, solubilized in urea, and folded; RPI-Ec, recombinant protein produced in E. coli and solubilized in urea; d, Ig-like domain; CTE, C-terminal extension; p, prime; b, boost; ip^h^, intraperitoneal inoculation with a heterologous strain; SP, signal peptide; IM, intramuscular; SQ, subcutaneous; PLGA, poly-lactic-co-glycolic acid; mRP-Pp, mannosylated recombinant purified protein produced in Pichia pastoris; dmRP-Pp, demannosylated recombinant purified protein produced in Pichia pastoris, NS, not significant.

*Chimeric constructs are shown with sequences separated by a slash (/). LigA and LigB domains included in constructs are numbered. An apostrophe (‘) is used to indicate that the endpoint of the sequence lies within a domain. Amino acid coordinates are shown in parentheses.

** All experimental trials in a study may not be shown. In those cases, a representative trial is shown.

An important aspect of the 2004 Koizumi and Watanabe study was immunization with recombinant Lig proteins expressed as His6 fusion proteins for purification purposes. Expression of such large (~120 kD), recombinant Lig proteins has proven to be difficult for other laboratories to produce in soluble form. A subsequent vaccine study, published in 2006 by the group at Cornell University, involved expression of recombinant LigA fragments as glutathione-S-transferase (GST) fusion proteins to enhance solubility. Two recombinant LigA fusion proteins were produced; the first protein consisted of the region of LigA largely though not entirely shared with LigB (domains 0-7’, **Figure 1**), while the second protein consisted of the region unique to LigA (domains 7’-13, **Figure 1**) (122). Both the shared and unique regions were combined and adsorbed to the adjuvant aluminum hydroxide (Al(OH)_3_) for immunization purposes. A weakness of the 2006 study was that the serovar Pomona challenge strain required inoculation at a dose of 10^8^ organisms for 50% of hamsters to reach endpoint criteria (ED_50_). Because hamsters were challenged at the ED_50_ dose, a statistical difference between groups of control- and LigA-immunized hamsters was not observable based on endpoint criteria alone (**Table 1**). Nevertheless, the LigA-immunized hamsters had a significant advantage in terms of the severity of renal histopathology.

Subsequently, efforts were made to identify the region of LigA responsible for immunoprotective activity. A study published in 2007 involved immunization with Freund’s adjuvant combined with recombinant His6-tagged LigA expressed in *E. coli*, purified under denaturing conditions and then refolded by stepwise dialysis. The initial immunization was performed with Complete Freund’s Adjuvant (CFA), a water-in-oil emulsion containing killed mycobacteria (123). Specific mycobacterial cell wall components in CFA, *N*-glycolyl muramyl dipeptide and the glycolipid trehalose-6, 6 dimycolate, stimulate cellular immunity and antibody production and induce a mixed Th1 and Th17 response (124–126). Subsequent immunizations were performed with incomplete Freund’s adjuvant (IFA), which is CFA without mycobacteria. IFA induces a balanced Th1/Th2 response and produces an antibody response (125, 127). Immunoprotection was demonstrated with a recombinant His6 fusion protein consisting of the carboxyterminal region that is unique to LigA (LigA7’-13) (104). No protection was observed with the aminoterminal domains shared between LigA and LigB and carboxyterminal region of LigB. Subsequently, our group showed that this immunoprotective effect was found to reside primarily in LigA domains 10-13, with a minimum of three domains required for 100% survival of hamsters (109). LigA domains 11–12 were required but insufficient for immunoprotection, requiring combination with a third domain, either 10 or 13, for immunoprotection.

Because of concern for the safety of Freund’s adjuvant, LigA7’-13 was also tested with the adjuvant aluminum hydroxide (Al(OH)_3_), which is used in several FDA-approved vaccines (128). Aluminum hydroxide is comprised of nanoparticles that form porous aggregates to which the antigen adsorbs (129). Aluminum hydroxide maintains the antigen at a high local concentration in the depot to promote antigen uptake by antigen-presenting cells (128). “Danger” signals released from cells exposed to aluminum hydroxide stimulate APCs, eventually leading to a Th2 response for most antigens (130, 131). In contrast to the results with Freund’s adjuvant, significant immunoprotection against *L. interrogans* was not observed in four studies with LigA7’-13 adsorbed to aluminum hydroxide despite generation of specific IgG responses (**Table 1**, Fisher’s Exact Test) (106, 110, 111, 116). Given the uptake and survival of *L. interrogans* in macrophages in cell culture and *in vivo* models (64, 65), one possible explanation for the advantage of Freund’s adjuvant over aluminum salts is that immunoprotection from *L. interrogans* involves Th1 lymphocyte activation of macrophages with intracellular leptospires. In addition, both Th1 and Th17 cells were involved in protective immunity against other predominantly extracellular bacteria in immunoprotection studies (132, 133). One study that did observe immunoprotection with aluminum hydroxide involved mannosylated LigA7’-13 synthesized in the yeast *Pichia pastoris* (113). Mannoproteins are targeted to DC-SIGN and mannose receptor on the surface of antigen presenting cells (APCs) to enhance antigen presentation (134).

Modern adjuvants known to induce a more robust Th1 response were also tested. Biodegradable particulate systems, such as liposomes and microspheres, enhance delivery of protein antigens to APCs (135, 136). In one study, hamsters were protected from illness following immunization with LigA7’-13 that was packaged in a phosphatidylcholine-based liposome for immunization (106). The animals vaccinated with the liposome formulation had a higher titer of anti-LigA IgG than those vaccinated with aluminum hydroxide as adjuvant, which was not significantly protective (106). Similar protection was obtained by the same group when poly-lactic-co-glycolic acid (PLGA) microspheres were used as carrier for LigA7’-13, although the antibody titer was similar to when aluminum hydroxide was the adjuvant (**Table 1**, Fisher’s Exact test). In general, PLGA particles cause a Th1 bias in the immune response (136), although splenic Th1 and Th2 cytokine mRNAs were both increased following immunization with the LigA7’-13/PLGA particles relative to transcript levels from hamsters immunized with LigA7’-13/Al(OH)_3_ (106). In another study, neutral liposomes were mixed with the saponin QS-21 and the TLR4 agonist monophosphoryl lipid A (120, 121). 60% of the immunized hamsters survived challenge, whereas no animals immunized with adjuvant alone survived (121). Altering the physicochemical properties of the liposome such as its surface charge or size may substantially improve performance of liposome-based vaccines (137). On the other hand, LigA7’-13 was not protective when introduced into hamsters using carboxyl multi-walled carbon nanotubes (COOH-MWCNTs), even when CpG oligodeoxynucleotides, a potent TLR9 agonist, were added to the carrier (116). Similarly, CpG oligodeoxynucleotides (ODNs) had no effect as an adjuvant for LigA7’-13 in a separate study (114). Because the sequence of the CpG ODNs was optimized to stimulate mice and human TLR9 (138), and the optimal CpG ODN sequence for stimulating hamster TLR9 is unknown, the negative results do not rule out the possibility of TLR9 promoting immunity to *L. interrogans* in hamsters. Finally, different polysaccharides have been tested as immune adjuvants with other pathogens (139). Xanthan, an exopolysaccharide of *Xanthomonas* spp., exhibited strong immunoprotection against *L. interrogans* when used as an adjuvant for LigA7’-13 (114).

An alternative approach that would be appropriate for immunization of reservoir hosts is to present LigA7’-13 as an oral vaccine. We have shown that hamsters could be moderately protected against intradermal challenge by oral immunization with *E. coli* expressing a lipidated form of LigA7’-13 (112). Protection from intraperitoneal challenge did not reach statistical significance by Fisher’s Exact test (**Table 1**). The antigen was expressed from a plasmid encoding the OspA signal peptide fused to LigA7’-13. Triacylated lipopeptides stimulate signaling through TLR1/TLR2 heterodimers (140), and the lipid moiety influences the appearance of specific IgG subtypes in mice orally immunized with *Lactobacillus plantarum* expressing N-terminal triacylated OspA (141). *L. plantarum* is being explored as a vehicle to orally deliver leptospiral immunogens (142) in part because of its ability to stimulate innate immune memory *via* macrophages (143).

Considerable effort has been devoted to development of a LigB vaccine because *ligB* is found in, if not expressed by, more pathogenic *Leptospira* species than *ligA* (34). Neither LigB7’-12 as a recombinant protein with Freund’s or aluminum hydroxide adjuvant nor DNA with aluminum hydroxide was protective (104, 107, 111). In addition, DNA encoding the C-terminal extension of LigB was not protective (111) (**Table 1**). Immunization with the early Ig-like domains of LigB shared with LigA as recombinant protein has conferred varying degrees of protection. As shown in **Table 1**, some studies have reported largely negative results (104, 108, 118), another moderate success (107), and a more recent study nearly complete protection (117). The success of the latter study may be accounted for by the use of an intramuscular route of immunization instead of the subcutaneous route used for the other studies (**Table 1**). However, it should be noted that in a separate study, hamsters immunized with LigB0-7' protein via the intramuscular route were not protected from heterologous challenge (115) (**Table 1**).

Published Lig vaccine studies have employed a variety of approaches to evaluate protection results including antibody responses, lymphoproliferative responses, histopathology, as well as the number of animals that met endpoint criteria. Periodic acid Schiff staining and a histopathology scoring system are particularly helpful in evaluating the impacts on vaccination on renal histopathology (109). Current approaches to examining cellular immune responses involve measurement of Lig-specific lymphoproliferative responses of splenic cells and determination of cytokine mRNA levels in vaccinated hamsters (105–107). A preliminary determination of the T helper response can be made based on the IgG isotype generated by vaccination. Hamster IgG isotypes involved with immunoprotection have been determined (116, 117, 119, 121), yet the T helper responses associated with the IgG subsets have not been well characterized in hamsters. Experiments in mice may be more informative due to the well-characterized immune system and the available reagents. Statistical evaluation of subtle outcome differences in small sample size (i.e., fewer than 30 animals) studies using binary (pass/fail) endpoints by Fisher’s Exact test is often less sensitive than comparison of survival curves using the Log-rank (Mantel-Cox) test. For example, while the difference between the group immunized with LigBCon vs. the control group of 2/6 vs. 0/6 in the study by Cao et al. (108) is not significant on a simple binary analysis, the survival curves are significantly different at a level of P < 0.05 by Log-rank analysis.

An advantage of a protein-based vaccine against leptospirosis is the potential for broad, cross-protection against multiple species and serovars. Cross-protection with Lig proteins seems possible given their relatively high degree of sequence conservation. For example, we found 96.4% average LigB amino acid sequence identity among eleven different *L. interrogans* and *L. kirschneri* serovars (**Table 2**). Most current leptospiral vaccines consist of inactivated whole bacterial cells and elicit a humoral response primarily against LPS, whose structure differs considerably among leptospiral serovars. As a result, immunity generated by whole cell vaccines is generally serovar specific. For this reason, whole cell vaccines typically contain a combination of serovars prevalent locally (144). In some studies, heterologous protection against unrelated serovars has been observed (145, 146), including cases with altered or depleted LPS (147–149). As discussed in a recent review on leptospirosis vaccines (150), these varying results may also stem from different levels of immunoprotective protein antigens, such as the Lig proteins, whose expression varies according to the culture conditions used to grown the antigen source or challenge strain.

**Table 2:** Comparison of LigB sequences from 11 serovars* of *L. interrogans* and *L. kirschneri*.

	Percent identity		
	Minimum	Maximum	Mean
LigB	93.00	99.42	96.41
LigB 0-7’	91.51	99.65	95.96
LigB 7’-12	93.61	100.00	97.33
LigB CTE	92.61	100.00	96.61
LigB 10/7/7**	95.83	100.00	97.96

*L. interrogans serovars Icterohaemmorhaegiae strain Verdun (accession number EKP20873), Lai 56601 (AAN50976), Australis B0192, Manilae UPMMCNIID (AKP27178), Canicola (POR18499), Pomona UT364) EMO00594), Pyrogenes 200701872 (EMP05929), Hardjo (ALN99229), Bratislava (AKH76029), Copenhageni Fiocruz L1-130 (AAS69085); L. kirschneri serovar Grippotyphosa RM52 (AASP04736).

**A single chimeric Ig-like domain comprising N-terminal sequences from LigB10 and C-terminal sequences from LigB7.

There is some experimental evidence that LigB can mediate heterologous protection. Cross protection was observed in one study with a DNA vaccine encoding the Ig-like domains shared between LigA and LigB (LigB0-7’) from serovar Canicola of *L. interrogans* (111). DNA vaccines have the potential of stimulating both an antibody and a Th1-biased response (151), which may be an advantage given the evidence for intracellular invasion of macrophages (38, 65). Five of eight hamsters immunized with the DNA with aluminum hydroxide were protected from lethal challenge with serovar Copenhageni, whereas none of six control hamsters survived (*P* = 0.03). However, these results may not be robust. In a later study only two of five hamsters immunized with the same DNA vaccine were protected versus none of five in the control group (*P* = 0.44) (115). Homologous challenge with a Canicola strain that would have revealed the maximum possible protective effect with the immunization regimen was not performed in either study. A prime-boost regimen exhibited more promising results in the same study. Prime boost regimens have the potential to broaden the immune response, as demonstrated for influenza vaccination (152). Hamsters were first immunized with the DNA vaccine adsorbed to aluminum hydroxide and then boosted with recombinant LigB0-7’ protein adsorbed to the adjuvant 21 days later. Specific IgG levels were higher in animals subjected to the prime-boost regimen than those that received the DNA vaccine for both immunizations, although lower than the group that received the recombinant protein for both inoculations (115). Only the group receiving the prime-boost was significantly protected from heterologous challenge with the Copenhageni serovar (5 of 6 survivors) (115). None of the five animals immunized with the recombinant protein alone were protected despite the high levels of LigB antibody produced by the immunization.

Critical to any evaluation of any candidate leptospirosis vaccine is its ability to not only prevent overwhelming infection and endpoint criteria such as lethality but also residual renal infection among survivors. Prevention of sublethal infection is particularly important for vaccines intended for animals. LigA7’-13 vaccines have typically revealed sublethal renal infection regardless of adjuvant, as determined by culture, serology, histopathology and/or real-time PCR. Even in studies showing dramatic 100% vs. 0% survival rates in Lig- vs. control-immunized animals, burdens of infection up to ~1 million copies of leptospiral DNA per microgram of hamster tissue DNA have been documented in hamsters surviving infection (109). In contrast, recent studies that have shown protection from lethal infection with domains shared by LigA and LigB as protein (117) or DNA (111) showed culture negativity of kidneys from most survivors in the vaccinated group. The immunological mechanisms necessary for sterilizing immunity are unknown, but several observations suggest that renal production of IFN-γ is involved (153, 154). Mice deficient in IL-10, a negative regulator of IFN-γ production, rapidly clear *L. interrogans* from their kidneys (155). In addition, a whole-cell serovar Hardjo vaccine that prevents renal colonization in cattle stimulates a potent Th1 response; CD4^+^ and γδ T cells from the animals proliferate and produce IFN-γ in response to Hardjo antigens (156).

In a structure-based approach, investigators took a rational approach to vaccine design by first considering the low-resolution structure of the tandem Ig-like domains of LigB (29). The extensively accessible surface area involves almost all of the 12 domains, justifying their subsequent effort to generate monoclonal antibodies against the entire length of the 12 Ig-like domains of LigB. Knowledge of the NMR-derived structure of LigB12 (26), which was assumed to be similar across all 12 Ig-like domains, allowed them to finely map the epitopes of bactericidal monoclonal antibodies to specific surfaces of a domain. Surfaces targeted by highly bactericidal monoclonal antibodies were combined to form a single chimeric Ig-like domain. The chimera comprised β strands A, B, B’, and C from domain 10, β strands C’, D, E, and F from domain 7, and β strands G and G’ from domain 7. This “LigB10-B7-B7” chimeric protein completely protected hamsters from lethal challenge with *L. interrogans*, whereas all hamsters immunized with LigB7, LigB10, or PBS succumbed to the infection. The bacterial loads in the liver, kidney, and bladder of animals immunized with the chimera were orders of magnitude lower than in tissues from animals mock immunized with PBS. No histopathological damage was observed in animals immunized with LigB10-B7-B7. These promising results demonstrate the advantages of structure-based vaccine design for guiding construction of leptospiral vaccines based on recombinant surface proteins.

## Conclusions

**Figure 7:**
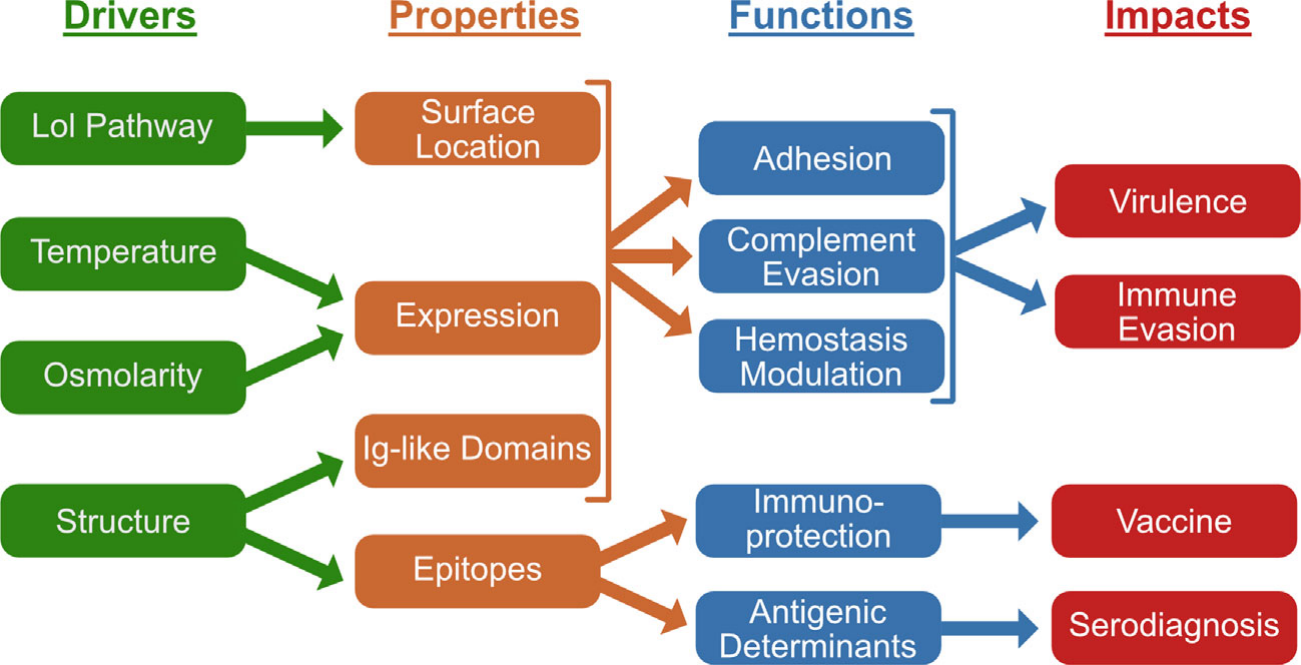
Summary of Leptospiral Immunoglobulin-Like (Lig) Protein Drivers, Charateristics, Functions, and Impacts. Lig proteins are exported to the leptospiral surface as lipoproteins *via* the localization of lipoprotein (Lol) pathway. Expression is stimulated by temperature and osmolarity. Their immunoglobulin (Ig)-like domains are responsible for various functions including leptospiral adhesion, complement evasion and modulation of hemostasis. These functions contribute to leptospiral virulence and immune evasion. Lig protein epitopes have been found to contain immunoprotective vaccine and serodiagnostic determinants.

*Leptospira* speciesbelonging to the P1 and P2 clades contain one or more members of the family of surface-exposed, virulence proteins containing a series of Ig-like domains: LigA, LigB, and LigC. The various drivers, characteristics, functions, and impacts of the Lig proteins covered in this review are summarized in **Figure 7**. As indicated by studies involving simultaneous transcriptional knockdown of *ligA* and *ligB*, some or all of the roles played by these proteins appear to be required for infection of hamsters. While expression of LigA and LigB is strongly induced by osmolar and thermal conditions found in the mammalian host, little is known about the regulatory pathways controlling their expression. The Lig proteins interact with a range of host proteins and appear to play multiple roles in host invasion and evasion of innate immunity, including complement resistance and hemostasis. Despite the surface accessibility along the length of the Ig-like domains, almost all host protein interactions are confined to the C-terminal Ig-like domains that differ between LigA and LigB. The structure and function of the putative Big_5 domains in the LigB C-terminal extension remain to be investigated beyond their involvement in fibronectin binding. LigB is a logical target for vaccine development because its gene is present in most pathogenic species of *Leptospira*. However, protective immunity accompanied by dramatic reductions in the burden of infection in the kidneys has been achieved in few studies. Future success of vaccine development with LigB will require a better understanding of the cellular immune mechanisms associated with immunoprotection. While the Lig proteins have been examined extensively since their discovery nearly two decades ago, their potential as vaccines and serodiagnostic antigens for prevention and diagnosis of leptospirosis remain to be fully realized.

## Author Contributions

DH and JM conceived the review, appraised the literature, and wrote the manuscript. All authors contributed to the article and approved the submitted version.

## Funding

The work was supported by a Veterans Affairs Merit Award and a National Institutes of Health grant R21AI128560 to DH.

## Conflict of Interest

The authors have relevant intellectual property related to the Lig proteins.
